# Supplementary material for: The discovery of novel noncoding RNAs in 50 bacterial genomes
Source: Nucleic Acids Res. 2024 Apr 22;52(9):5152–65. doi: 10.1093/nar/gkae248 (PMC11109978; doi:10.1093/nar/gkae248)
Supplement: gkae248_Supplemental_Files [file gkae248_supplemental_files.zip › Narunsky 50 Genomes RNA Motifs Files.zip!/Publication/66-P_ruminicola/Figures/LRC-66-24-NC_014033_1_IGR_63_1085416_1085590.sto.pdf]

LRC-66-24-NC_014033_1_IGR_63_1085416_1085590.sto.r2r

5′-○○○○RCAAGACAGAAGCGCGUCAGCUUCGUUU●C●AA●ARRAURCUGCRGA●GCACR●AUGCA●GRGCAGYARGARYRRYRAYGRCY●●U●YA●Y●C●●UYRUUCYGGGUGCAAAGAUACGAA●●A●UUYCGAAU●●RCRR●AAUU●Y●●GRAAAA
